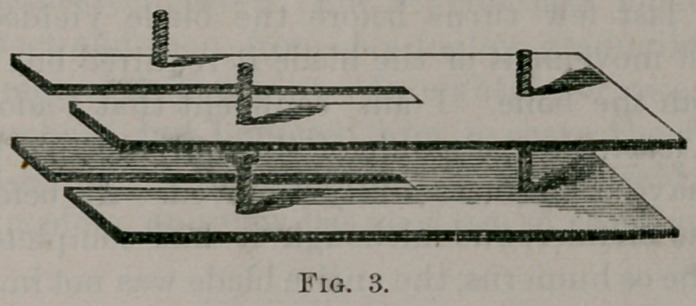# Two Remarkable Surgical Cases

**Published:** 1884-06

**Authors:** W. F. Westmoreland

**Affiliations:** Professor of Surgery in the Atlanta Medical College, Atlanta, Ga.


### TWO REMARKABLE SURGICAL CASES.

BY W. F. WESTMORELAND, M. D.

Professor of Surgery in the Atlanta Medical College, Atlanta, Ga

The object of this paper is, to place on record two rare surgical cases which presented themselves to me for treatment during the past winter, and which at the time greatly interested me as well as those who assisted me in their treatment. We feel that they will not be without interest to the profession, if for nothing else than their rarity and peculiarities:

### CASE FIRST.

On the 28th of November, 1883, S. S., seventeen years old, of Louisville, Alabama, presented himself at the surgical clinic of the Atlanta Medical College for treatment. The following history of his case was obtained from his father who accompanied him to the city: Five years ago, when the boy was only twelve years of age, he rose from his bed before daylight to build the morning fire, and in approaching the fireplace fell over a chair and landed with his face in the fireplace, in the midst of the smouldering coals and embers of the fire of the preceding night. His mouth and its vicinity appears to have received the most extensive burn, greater upon the left than the right side, as will be seen from wood-cut, Fig. 1. It would appear from the cicatricial tissue and the extent of its contractions, that the borders of the lips were entirely destroyed. The burn also extended to the orbicular muscle, and in more than one point destroyed almost the entire thickness of the muscle, as well as the fascæ and other tissues in the vicinity. There was such destruction of the tissues of the mouth and such contraction of the cicatricial tissue, as to leave a very small opening to the buccal cavity, not by any means so large as would be suggested by the wood-cut; the opening barely admitting, with considerable force, a No. 10 bougie. For two to three inches in every direction, as will be seen by Fig. 1, cicatrices cover the skin. Only at certain points did the burn destroy the entire thickness of the true skin, but sufficiently numerous to produce the contraction and terrible deformity so well shown in the wood-cut. As will be seen, the destruction of tissue extended to the skin in the vicinity of both eyes, roducing ectropion of the inferior lids of both eyes, much worse upon the left than the right side, as is well demonstrated in Fig. 1. The father stated that but little had been done to remedy the defect, in fact nothing more than an effort to dilate the small opening to the buccal cavity with bougies and dilators and an occasional slight incision to permit more freely their introduction. The object of this temporizing treatment was to keep the opening sufficiently large to introduce into his stomach a sufficiency of nourish-

FIG. 1.

ment to sustain him. But it was readily seen from his emaciation, pallor of skin, with all the marked symptoms of anemia that presented themselves, that he did not receive a sufficiency of nourishment, and that if left to himself, without any attempt at artificial feeding, he would not last a great while. As there was no possibility of entering the mouth with anything larger than a number ten bougie, but little could be determined as to the actual condition of the buccal cavity. I found it impossible to satisfy myself as to the existence or absence of adhesions between the lips and cheeks and the soft parts covering the superior and inferior maxillary bones, or to estimate the condition of the mucous membrane covering the lips and cheeks, whether in a normal state, partially destroyed, and to what extent. It was evident that this could not be determined until an incision was made sufficiently extensive to introduce the finger and thus reveal those important facts.

The question to solve was how best to make an opening of the mouth that would remain permanent, and not be subject to the contraction of the cicatricial tissue that occupied the point of the original mouth. If this could not be done, however extensive the incision, the contraction of the inodular tissue which would, to some extent, be added to by the incision, would sooner or later contract the orifice, and we would have as the result the same condition that now exists. It is true, it might be retarded and even prevented from reaching the point it is now, by a rigid system of dilatation with an occasional incision, but then it would have to be continued during life, with all its annoyances, suffering, and with the continuance of the shocking deformity.

Without hesitation, I determined, although tedious and trying, both to the patient and myself, to adopt a course which, if successful, would give permanent relief, and would, I had hope, relieve, to a very great extent, the hideous deformity then existing. The plan proposed and which I later adopted, was to make an incision to the extent of a few lines beyond the size of a normal mouth and as near the normal position of the mouth as I could judge, with the hope that I would find the mucous membrane lining the lips and cheeks sufficiently normal to be dissected up to an extent to transplant or rather slide over the lips of the incision, or rather the lips of the mouth made by the incision, and by fitting accurately the mucous membrane in position, to make the mucous border of the lip; in other words, to line the entire incision, the raw surfaces making the lips with normal mucous membrane, and secure it in position by sutures, pins, plaster, etc, with the hope of union. If successful, we would have an opening lined with mucous membrane which would not contract and thus make a permanent opening or mouth. The extent that the deformity would be relieved would certainly depend upon the care that was taken in modeling the mouth, and in attaching the mucous membrane to imitate the border of a normal ip.

After getting my patient in the best possible condition by rest and an extra quantity of nutritious food, I partially etherized him, and after fixing him securely in a semi-reclining position, I made an incision upon either side of the small orifice, sufficiently extensive to make an opening large enough to readily admit the introduction of the index finger, and a thorough exploration of the buccal cavity. To my great joy I found the mucous membrane of the cavity in a much more normal condition than I expected. I found only three slight adhesions of the mucous membrane of the lip and cheek and that covering the superior maxillary bone; one on either side in the vicinity of the bicuspid teeth, and one in front and just above the incisors. The incision was now extended until it was a few lines larger than a normal mouth would have been. With a strong pair of scissors, the adhesions of the lip and cheek to the bone were completely detached, so that both were readily movable in any direction. After arresting the hemorrhage by means of pressure and a solution of carbolic acid, I made a careful inspection of the character of the tissue through which my incision was made, and found that very nearly the whole extent was cicaticial tissue extending upon both lips at points several lines in extent. The mucous membrane I found in a sufficiently normal condition to answer my purpose. I then commenced dissecting up the mucous membrane from the lips and cheek. At some points it was detached to the extent of half to three-quarters of an inch, while at other points it extended an inch or more. The extent of the dissection was necessarily dependent upon the extent and location of the denuded lip to be covered by the detached mucous membrane. When this was sufficiently dissected up, it was not only necessary to trim the detached mucous membrane but to trim the lips of the incision, removing to a great extent the inodular tissue, and so shaping the lips that when the mucous membrane was drawn over them, to assume the shape of a mouth. When all was completed and the blood ceased to flow, the parts were carefully cleansed with a solution of carbolic acid, and with a delicate needle and the smallest silk suture carbolized, the detached mucous membrane was brought accurately into place and secured by numerous interrupted sutures. Certainly it required an occasional nip with a pair of scissors or a touch with a bistoury to still further detach the mucous membrane, and pare the lips to have it fit accurately and without too much tension. After the stitching was completed and the parts thoroughly cleansed and carbolized, they were still more firmly secured by the finest isinglass plaster.

It was certainly a fatiguing and tedious operation, requiring three hours or more to perform it, but the results fully compensated both my patient and myself for the fatigue, trouble and pain endur-

FIG. 2.

ed. At the end of the second day the isinglass plaster was to a great extent removed and every suture that appeared giving the least irritation was removed, the parts cleansed and carbolized and the plaster reapplied. On the fifth day after the operation, the dressing was all removed and it was found that all had united by the first intention save a small point a few lines in extent. The sutures were now all removed and the parts thoroughly cleansed with carbolic acid solution, and the isinglass dressing reapplied.

After this an occasional dressing with the plaster for a week or ten days longer constituted the treatment.

In trimming the lips of the wound made by the incision and which ultimately made the mouth, I found at two points the almost complete destruction of the orbicularis oris muscle. To the extent was this muscle destroyed that I feared there would ever be very great defect in controlling the lips and movements of the mouth. Soon after the union of the edges of the wound, there was no motion of the lips and mouth ; the parts were immovably fixed in their position. In a short time, however, slight motion was perceptible, which gradually increased, to the extent that when the photograph was taken for wood-cut, Fig. 2, you could see that he had recovered the action of the muscles of the vicinity to the extent that you could readily recognize a smile upon the countenance and could actually control the orbicularis oris muscle to the extent of producing the sound of whistling. I feel confident that he will ultimately recover almost the complete functions of the muscles in the vicinity of the mouth.

To show the extreme condition in which he presented himself I desire to call attention to the fact that just so soon as the opening was made which permitted him to take a sufficient amount of nourishment, his haggard appearance, with all the symptoms of anemia disappeared and he rapidly gained flesh as will be readily seen in wood-cut, Fig. 2.

After a rest of two or three weeks I turned him over to the eye and ear clinic, and Dr. A. W. Calhoun performed the ordinary operation for ectropion of the left lid with the beautiful results as shown in the wood-cut, Fig. 2.

(Knife impacted in shoulder joint)

CASE SECOND.

On January the 16th, 1884, Willie Johns, aged 16 years, was brought to my office for treatment. Upon investigation I learned the following particulars of his case: In a fight with another boy, near his own age and size, he received a knife wound, the blade passing through the deltoid muscle and penetrating the os humerus a few lines external to the bicipital groove, passing through the great tuberosity near the articular surface and transfixing the head of the bone so that when the bone was moved the point of the knife blade could be felt in contact with the glenoid cavity of the scapular—thus at every movement of the os humerus wounding the senovial membrane and other soft parts lining this cavity, as well as doing injury to the cartilage and bone itself. The blade was apparently immovably fixed in this position with the handle of the knife intact. The strange and hardly to be believed part of the history of the injury was, that the knife was not driven through the bone when it was in the hand of his antagonist but was thrown a distance of ten feet or more by his antagonist. This I was not disposed to believe when first stated by the patient, but all who witnessed the fight as well as the two combatants persist in the correctness of the statement.

Before he reached my office numerous efforts had been made by professional gentlemen, as well as others, to remove the blade; the handle being intact with the blade, gave a good hand hold so that with the humerus held in position the whole strength of the man making the effort could be exerted under the most favorable circumstances for the extraction of the blade. After reaching my office the effort was repeated under the most favorable circumstances without the least impression on the blade. The blade penetrated the bone transversely, the edge of blade presenting outwards with the back inwards. I now determined to try another plan, that of short and quick oscillating motions from within outwards so as to increase the size of the wound in the bone, but after a half hour or more of constant effort it was evident that the blade had not moved the hundredth part of a line.

The question that presented itself now was what could and should be done? If the blade was broken off and left in position we could expect nothing but an acute suppurative inflammation of the shoulder joint, with its complete disorganization, requiring a resection at a time when it would endanger the life of the patient. Should we resect now either partially or completely the head of the bone, or should we attempt some other mode of removing the blade from its apparently fixed position. I decided to try some other mode of extracting it. One of the great difficulties in attempting to arrange for its removal was the fact that the blade was narrow and long, and any force that was applied not in the direction of the axis of the blade sufficiently great to remove it would certainly result in breaking the blade and thus bring us face to face with a serious surgical operation—that of a partial, or complete resection of the shoulder joint.

After sending around to the various instrument depots, silversmiths and machine-shops, I could get nothing that I felt would be safe in attempting the effort of extraction. All this required two or three hours, while the brave boy lay quietly on his couch without a murmur. After finding that I could not improvise anything that I regarded as safe to attempt the removal, I determined to have one constructed. After all the instrument-makers and silver-smiths and mechanics refused to attempt anything until the next day, I appealed to an ordinary blacksmith near my office, who at once went to work and, in two or three hours, made the instrument, the wood-cut of which is represented in Fig. 3. While it is rough and without polish, still it filled the indication, as will be seen, most perfectly. This elevator, as I call it, as will be seen from the wood-cut, consists of two plates, with a slot in the centre of each, running two-thirds the length of the plates and about the eighth of an inch wide. To the bottom plate there is solidly attached three bars with screws cut their whole length. The top plate is pierced by three openings

FIG. 3.

corresponding to the three bars in the bottom plate and for their reception. On each bar there is a nut, with screws cut fitting the screws cut on the bars. When the nuts were screwed down to the bottom plate, the top plate followed it, so that we had the plates only separated by the nuts; from the bottom of the bottom plate, to the top of the upper plate, would be about a third to half an inch. When the plates were in this position the instrument was applied to the knife-blade, which was readily done as the blade was forced into the slot of the plates and the plates then pushed up until the blade rested midway between the two bars and nuts in front, and the one nut and bar behind. The slot embraced the blade just below the jaws of the handle of the knife. To separate the plates now, with the lower plate resting upon the os humerus and thus fixing it, with the upper portion or shank of the blade and jaws of the handle of the knife fixed in the slot or rather above it, of the upper plate, would necessarily lift the blade from its apparently fixed position, unless something should break, but as the lifting was in the direction of the axis of the blade, there could be but little or no danger of breaking the blade. With a wrench that fitted the nuts they were gradually separated from the lower plate, carrying with them the upper plate with the knife attached to it.

The force necessary to start the blade from its imbedded position was immense, just how great it is certainly impossible to say. As an evidence of the force required, I would state that, after elevating the upper plate fully an inch, we found that the blade had not moved, but that the lower plate was imbedded in the soft parts, and while the skin had not given way, a few more turns of the nuts would certainly have lacerated the soft parts over the bone. To remedy this the nuts were turned back until I could raise the bottom plate sufficiently to introduce around it sheets of tin, thus making larger the base of the lower plate The nuts were again elevated and the force continued until the blade yielded. The force required was very great, as it required almost the strength of one man to move the nuts the last few turns before the blade yielded to the force. After the first movement of the blade it required but little force to remove it from the bone. I am confident that I adopted the only means, or at least, the principle of the only means, by which the blade could have been successfully removed. As before suggested, the blade was slender, and although it had completely transfixed the head of the os humerus, the entire blade was not imbedded in the hard and soft parts. The shank of the blade was the weakest part and the least deviation, as before suggested, of a great force from the axis of the blade would have resulted in breaking it at the shank, with all its unpleasant consequences.

After the blade was extracted the shoulder joint was immovably fixed and supported by means of a sling of moleskin adhesive plaster, passing under the elbow and over the opposite shoulder, with a second band of the same material passed around the body and over the injured os humerus, fixing it immovably to the chest. The wound was properly cleansed with carbolic solution, one to forty, and lint saturated with the same solution was applied to the wound and secured by means of adhesive plaster, with orders to keep it constantly wet with the carbolic acid solution. A decided opiate was then administered and the little sufferer was sent to his home. I had great fears that from the rough handling, and above all, the numerous wounds received by the soft parts covering the glenoid cavity by the point of the blade transfixing the bone, would result in an acute inflammation of the articulation, and, as above stated, took all precautionary measures to prevent it. On the day following the injury, he had considerable arterial excitement. Decided doses of opium were continued, and on the succeeding, or third day from the reception of the injury, the fever had subsided. and he was brought to my office by his father. I found the parts considerably swollen around the knife wound in the soft parts, which I attributed to the contusion of the lower plate of the instrument, as it was principally in the locality of the great pressure of the plate giving support or acting as a fulcrum to the instrument. The strips of adhesive plaster were slightly loosened, and the same dressing was reapplied and ordered to be kept damp with the carbolic acid solution as before.

No unpleasant symptoms presented themselves, and at the expiration of ten days the wound had healed, and all swelling and soreness in the vicinity of the shoulder joint had subsided, and he was becoming restive under the restraint of having the limb fixed by the moleskin adhesive plaster. For several days longer I retained the plaster intact and then, from day to day, gradually loosened it, giving slight motion to the joint. In eighteen or twenty days the plaster was removed, but I ordered him to carry the fore-arm in a sling and not to use it. He recovered, as before said, without an unpleasant symptom, and now has good use of the shoulder joint.

## Figures and Tables

**Fig. 1. f1:**
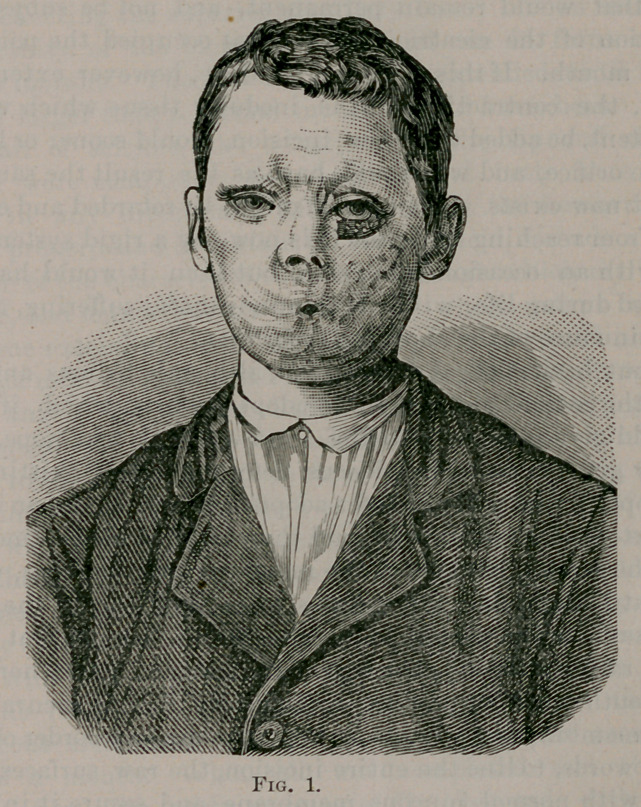


**Fig. 2. f2:**
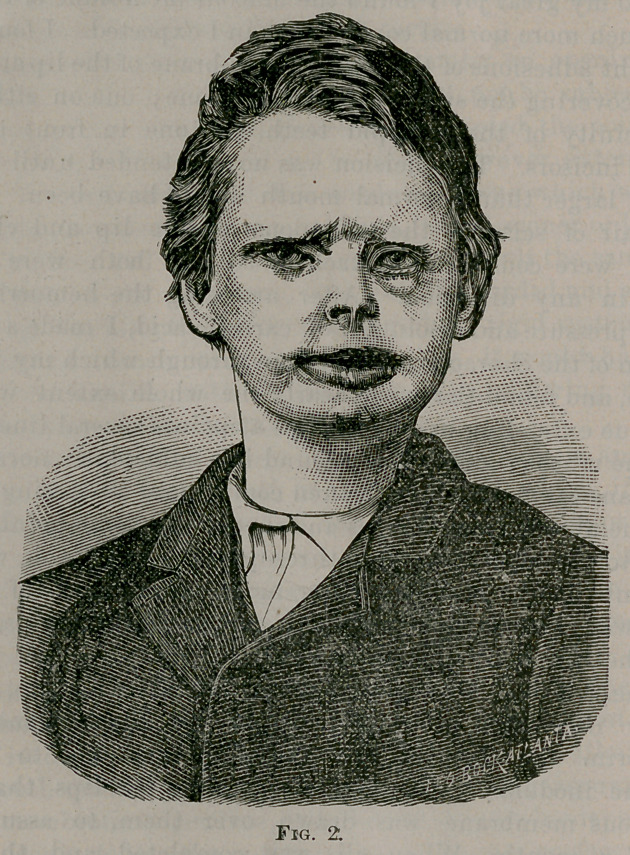


**Fig. 3. f3:**